# Clinical Gait Evaluation with Neuromuscular Impairments (Clinical GENI) for spastic cerebral palsy

**DOI:** 10.3389/fnhum.2025.1637164

**Published:** 2025-09-17

**Authors:** Kylie Clewes, Yiwen Dong, Mary Meyer, Evan Lowe, Kornél Schadl, Jessica Rose

**Affiliations:** ^1^Motion & Gait Analysis Lab, Lucile Packard Children's Hospital, Stanford Medicine Children's Health, Palo Alto, CA, United States; ^2^Department of Computer Science, Stanford University, Stanford, CA, United States; ^3^Department of Orthopaedic Surgery, Stanford University, Stanford, CA, United States; ^4^Immed.AI, Budapest, Hungary

**Keywords:** cerebral palsy, gait analysis, neuromuscular impairments, muscle weakness, short muscle-tendon unit, impaired selective motor control, spasticity

## Abstract

**Background:**

Gait abnormalities in spastic cerebral palsy (CP) result from four primary neuromuscular impairments: muscle weakness, short muscle-relative-to-skeletal-length, muscle spasticity, and impaired selective motor control. The Clinical Gait Evaluation with Neuromuscular Impairments (Clinical GENI) was developed to help clinicians identify gait abnormalities and contributing neuromuscular impairments in spastic CP for use in any clinical setting.

**Aims:**

This study evaluated use and validity of the observational-based Clinical GENI to identify gait abnormalities and contributing neuromuscular impairments in children with spastic CP.

**Methods:**

Patients with spastic CP seen in 2023 for instrumented gait analysis and physical exam of neuromuscular impairments were evaluated using the Clinical GENI. Validity was assessed by agreement between identification of gait abnormalities on the Clinical GENI compared to 3D gait kinematics. Severity of neuromuscular impairments associated with gait abnormalities listed on the Clinical GENI was compared, and severity of neuromuscular impairment was correlated with severity of gait abnormalities.

**Results:**

Participants included 12 children with spastic CP (4 GMFCS I, 8 GMFCS II; mean age 11.25 years). The most common gait abnormalities were forefoot/flatfoot initial contact (IC) (16/24), flexed-knee IC (19/24), hip-flexion in single limb stance (12/24), and reduced pre-swing ankle plantarflexion (19/24). Strong agreement (83–100%) occurred between gait abnormalities on the Clinical GENI and kinematic values. Severity of neuromuscular impairment was higher (*p* < 0.05) for those with gait abnormalities vs. without and correlated to severity of gait abnormalities in a majority of comparisons.

**Conclusion:**

Results support clinical utility and validity of the Clinical GENI for evaluating gait abnormalities and contributing neuromuscular impairments in spastic CP.

## Introduction

Cerebral palsy (CP) is the most common movement disorder in children and has a higher prevalence among children born preterm (Oskoui et al., 2013). CP is diagnosed clinically based on delayed motor milestones, gait abnormalities, abnormal posture and tone, neuroimaging, and standardized motor and neurological testing (Wu et al., 2004; Zhou et al., 2017; Novak et al., 2017). There are three types of CP: spastic CP, dyskinetic CP, and ataxic CP, which arise from injury to different regions of the developing brain, and can co-exist. Spastic CP, the most common type, affects ~87% of children with CP and is associated with corticospinal tract (CST) injury of the developing brain (Sellier et al., 2016; Kitai et al., 2021). Dyskinetic CP affects 7–15% and is associated with damage to subcortical gray matter, including the basal ganglia and thalamus (Laporta-Hoyos et al., 2014; Himmelmann and Uvebrant, 2011; Kitai et al., 2021). Ataxic CP affects 4% of children with CP and is associated with cerebellar vermis injury or cerebellar malformations (Sellier et al., 2016; Kitai et al., 2021). The different types of CP have characteristic neuromuscular impairments and movement abnormalities associated with their specific regional brain injury. Accurate identification of neuromuscular impairments is critical to provide effective treatment for CP. Unfortunately, neuromuscular impairments are not often well-identified, compromising the ability to provide effective treatment (Ferrari et al., 2015). Consequently, research indicates current treatment outcomes are modest (Sees et al., 2020; Schwartz et al., 2022). Therefore, the Clinical Gait Evaluation with Neuromuscular Impairments (Clinical GENI) tool was developed to identify gait abnormalities and the contributing neuromuscular impairments in spastic CP in any clinical setting (Clewes et al., 2024).

The neuromuscular impairments of spastic CP arise from non-progressive CST injury occurring around the time of birth; however, the loss of functional mobility can progress (Bell et al., 2002; Sanger, 2015), especially as muscle growth fails to keep pace with skeletal growth leading to progressive joint contracture. There are four inter-related primary neuromuscular impairments which contribute to gait abnormalities in spastic CP: 1. muscle weakness, 2. short muscle-tendon units due to reduced muscle growth relative to skeletal growth, 3. muscle spasticity, and 4. impaired selective motor control (SMC) (Clewes et al., 2024).

The gold standard for evaluating gait abnormalities is 3D kinematic gait analysis (Gage and Novacheck, 2001) and should be considered when making surgical and non-surgical recommendations (Salem et al., 2024); however, 3D kinematic gait analysis may not be available to patients for a variety of reasons including cost or geographic accessibility. The Clinical GENI tool was designed to provide clinicians with a structured method for observational-based gait analysis and to draw connections to contributing neuromuscular impairments identified through physical exam, for use in any clinical setting. Advantages of The Clinical GENI tool include no additional cost, only requiring 2D video, and that it is more accessible and quicker to perform than 3D kinematic gait analysis. While 3D kinematic analysis provides precise measurements and is prioritized for treatment decisions and for evaluating treatment outcomes, the Clinical GENI tool was designed to support clinical evaluation to guide non-surgical treatments through identification of specific gait abnormalities and contributing neuromuscular impairments. The Clinical GENI is not an outcome measure or scoring tool designed to measure change over time. While there are other observational gait tools, such as the Edinburgh Visual Gait Score (Read et al., 2003) and the Observational Gait Scale (Mackey et al., 2003), these tools do not identify the neuromuscular impairments contributing to gait abnormalities and therefore, do not help focus targeted interventions to address specific neuromuscular impairments. The Clinical GENI tool does not address gait abnormalities directly related to skeletal deformity, leg length discrepancy, or balance impairments, which can occur in spastic CP. (van der Krogt et al. 2022) investigated neuromuscular impairments and their relationship to gait in CP, based on kinematic data with use of a computer program, but this does not use observational gait analysis that could be performed in any clinical setting.

The Clinical GENI tool was developed to identify common gait abnormalities observationally based on specific criteria and their associated neuromuscular impairments. Contributions of neuromuscular impairments to the gait abnormalities in the Clinical GENI were based on biomechanics, clinical expertise, and literature. The aim of this study was to evaluate the use and validity of the Clinical GENI tool to identify gait abnormalities and their contributing neuromuscular impairments in children and adolescents with spastic CP, in order to guide more targeted treatments that improve gait.

## Methods

All patients with a diagnosis of spastic CP who were seen in the Motion & Gait Analysis Laboratory at Stanford Medicine Children's Health from January 2023 through December 2023 were included in the study based on the following inclusion and exclusion criteria. Inclusion criteria were a diagnosis of spastic CP, age 3–17 y/o, ability to walk independently (i.e., without assistive device or external assist) for at least 6 meters, and had a physical exam of neuromuscular impairments including manual muscle test (MMT), passive range of motion (PROM), the modified Tardieu Scale (MTS), and the Selective Control Assessment of the Lower Extremity (SCALE) (Fowler et al., 2009). Exclusion criteria included a history of surgical intervention, including lower extremity (LE) orthopedic surgery or selective dorsal rhizotomy (SDR) within 1 year of gait analysis and/or botulinum toxin type A (BoNT-A) injection within 1-year of gait analysis, and no use of baclofen at time of testing. Gait abnormalities were identified using the Clinical GENI tool through video observation and also based on 3D kinematic data.

### Observational video analysis

The Clinical GENI video analysis tool originally reported by (Clewes et al. 2024), was refined by establishing specific criteria for each gait abnormality (Figure 1B in Supplemental material) and eliminating redundancy as neuromuscular impairments affecting loading response are the same for single limb stance and neuromuscular impairments affecting terminal swing are the same for initial contact. The tool lists common gait abnormalities seen in spastic CP, specific to each phase of the gait cycle. The clinician marks on the tool the presence of gait abnormalities for each limb and identifies the potential contributing neuromuscular impairments on the Clinical GENI based on findings from the physical exam (Table 1). The clinician can then focus treatment on those neuromuscular impairments.

**Table 1:** Demographics and physical exam of neuromuscular impairments.

**Demographics**		
Age (years) mean ± SD (range)	11.25 ± 4.27 (4–17)	
GMFCS (number of participants)	GMFCS I = 4; GMFCS II = 8	
CP diagnosis	Bilateral = 7; Unilateral = 5	
Sex	Male = 5; Female = 7	
History (Hx) of orthopedic surgery	Yes = 4 (GMFCS II = 4); No = 8 (GMFCS I = 4, GMFCS II = 4)	
Number of gait abnormalities per participant (mean)	Total 10.1 GMFCS I = 6.0, GMFCS II = 12.1, Bilateral = 13.4, Unilateral = 5.4	
Gait abnormalities per right side (mean)	Total 5.1 GMFCS I = 4.3, GMFCS II = 5.5	
Gait abnormalities per left side (mean)	Total 5.0 GMFCS I = 1.2, GMFCS II = 6.6	
**Physical exam**		
	**Right**	**Left**
**Muscle strength: Manual Muscle Test (MMT) score**		
Hip flexion (mean ± SD)	4.58 ± 0.53	4.73 ± 0.41
Hip extension (mean ± SD)	3.77 ± 0.62	3.66 ± 0.85
Hip abduction (mean ± SD)	3.58 ± 0.79	3.50 ± 0.70
Knee flexion (mean ± SD)	4.44 ± 0.76	4.08 ± 0.91
Knee extension (mean ± SD)	4.5 ± 0.45	4.63 ± 0.47
Ankle dorsiflexion (mean ± SD)	4.35 ± 0.98	4.60 ± 0.49
Ankle plantarflexor (mean ± SD)	2.00 ± 0.74	2.50 ± 1.17
**Muscle length: Passive Range of Motion (PROM)**		
Hip extension (°) (mean ± SD, COV)	−1.67 ± 10.73, 6.43	−4.58 ± 10.33, 2.25
Hip abduction (°) (mean ± SD, COV)	32.08 ± 10.33, 0.32	34.58 ± 7.53, 0.22
Straight leg raise (°) (mean ± SD, COV)	45.42 ± 13.05, 0.29	49.58 ± 12.52, 0.25
Knee extension (°) (mean ± SD, COV)	−2.08 ± 9.88, 4.75	−2.08 ± 9.40, 4.52
Ankle dorsiflexion, knee extended (°) (mean ± SD, COV)	−4.58 ± 12.15, 2.65	−5.00 ± 7.07, 1.41
Ankle dorsiflexion, knee flexed (°) (mean ± SD, COV)	6.67 ± 16.7, 2.50	10.00 ± 12.61, 1.26
**Spasticity: Modified Tardieu Scale (MTS) (R2-R1)**		
Rectus femoris (°) (mean ± SD, COV)	8.33 ± 21.25, 2.55	10.83 ± 21.93, 2.02
Gastrocnemius (°) (mean ± SD, COV)	4.58 ± 7.53, 1.64	2.08 ± 4.5, 2.16
**Selective motor control: Selective Control Assessment of**		
**the Lower Extremity (SCALE)**		
Total (mean ± SD)	5.58 ± 2.19	5.67 ± 2.67

**Figure 1:**
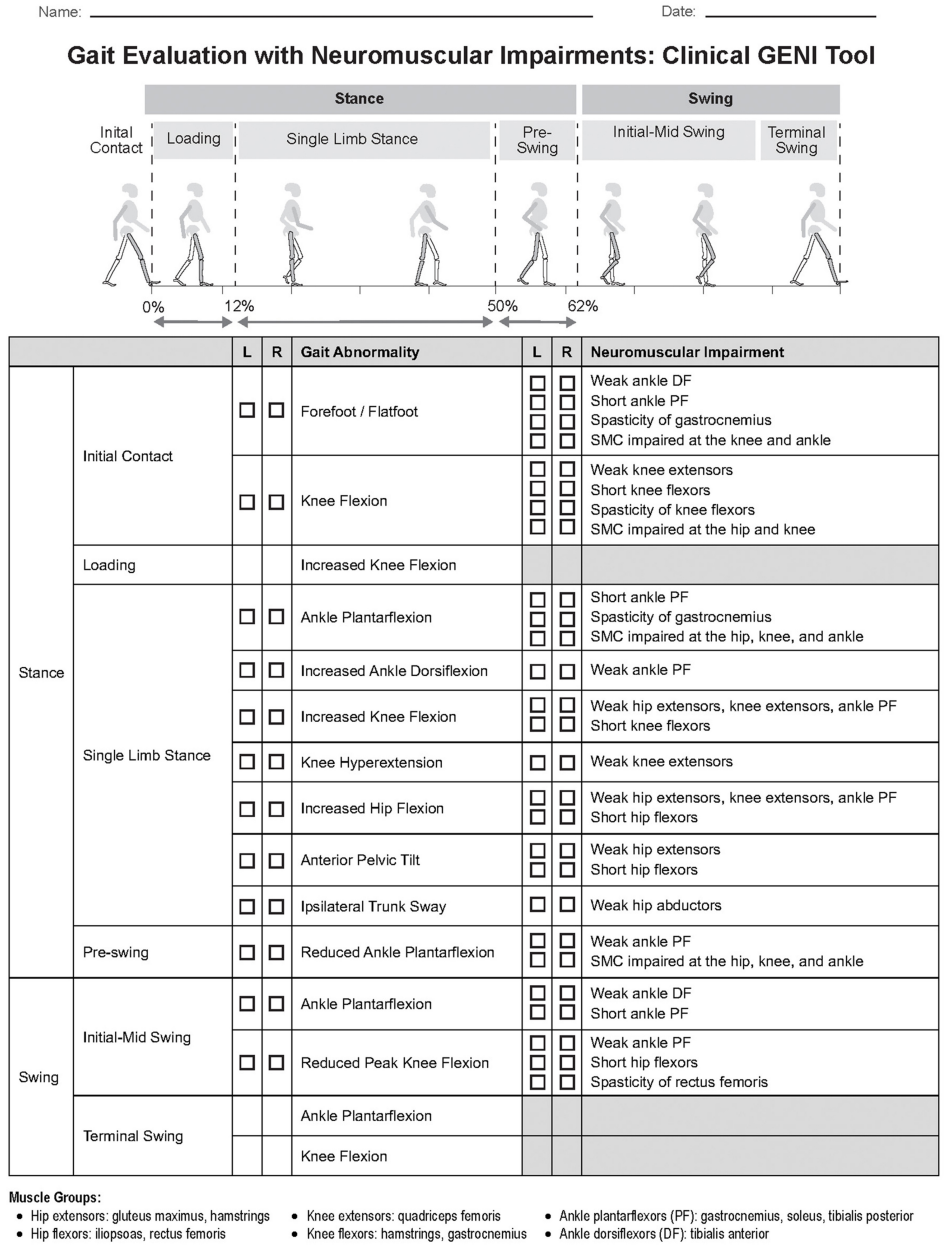
Gait evaluation with neuromuscular impairments: Clinical GENI tool. Figure 1b and (B) Clinical GENI tool criteria. Clinical GENI tool criteria is to be used with the Clinical GENI tool to determine whether a gait abnormality is present based on observational video analysis performed in the frontal and sagittalplanes (see Supplemental material for Figure 1B).

Two experienced gait lab physical therapists analyzed gait videos in the sagittal and frontal planes, that were recorded simultaneously with 3D motion capture, and used the Clinical GENI tool (Figure 1, Figure 1B in Supplemental material) to identify gait abnormalities on the right and left limbs over a minimum of 8 strides of walking. The evaluators were blinded to the physical exam and 3D gait analysis results while using the Clinical GENI tool. Observation of the ankle joint was performed through the talocrural joint to identify calcaneal plantarflexion in the presence of midfoot breakdown, which can be mistaken for increased ankle dorsiflexion (DF).

### 3D kinematic gait analysis data collection

Kinematic gait analysis was performed barefoot without use of assistive device or handheld assist along a 6 m path. Participants walked using self-selected speed. A modified Helen Hayes marker set was used with 18 reflective markers placed on the sacrum, the left and right anterior superior iliac spines, the anterior mid-shaft femurs, the lateral knee joint axes, the anterior mid-shaft tibias (shanks), the lateral malleoli, the calcanei, the dorsum of foot between the second and third metatarsal heads, the right and left acromia, and the left scapula. Four additional markers were placed on the right and left medial knee joint axes and medial malleoli. Reflective marker trajectories were recorded with an 8-camera optical motion capture digital system for 3D motion analysis (Motion Analysis Corporation, Santa Rosa, CA, USA) at a sampling rate of 100 Hz. OrthoTrak software version 6.6.4 (Motion Analysis Corporation Santa Rosa, CA, USA) was used to calculate joint kinematics from an average of three representative trials. Trunk, pelvic, hip, knee, ankle, and foot position during gait were used to calculate lower extremity joint kinematics for each limb.

### Physical exam

The physical exam was performed as part of the gait analysis test that included 3D gait kinematics and video to identify presence of and severity of neuromuscular impairments for children with spastic CP. For this analysis, the presence of and severity scores for neuromuscular impairments on the physical exam performed at the time of gait analysis were recorded and compared with severity scores for each specific gait abnormality for each participant. Neuromuscular impairments of muscle strength (MMT) (Manikowska et al., 2018), passive range of motion (PROM) (Sala and Ragni, 2024; Mutlu et al., 2007), spasticity (MTS) (Aloraini et al., 2022) and impaired SMC (SCALE) (Balzer et al., 2016; Fowler and Goldberg, 2009; Fowler et al., 2010) were based on physical exam findings at time of 3D kinematic gait analysis. These measurements are all included as National Institutes of Health (NIH) Common Data Elements (CDE) for CP. The MMT is rated as “Supplemental” for CP (National Institute of Neurological Disorders Stroke, 2024); Lower Extremity PROM is rated as “Supplemental” for CP (National Institute of Neurological Disorders Stroke, 2025); the SCALE is rated as “Supplemental” for CP (National Institute of Neurological Disorders Stroke, 2019); Tardieu Scale is rated as “Supplemental-Highly Recommended” for CP (National Institute of Neurological Disorders Stroke, 2020a); Instrumented Gait Analysis is rated as “Supplemental” for CP (National Institute of Neurological Disorders Stroke, 2020b). PROM was used to estimate muscle length with the knowledge that bony blocks or capsular tightness can confound the estimate of muscle length. For each participant, the severity score of each of the four neuromuscular impairments was recorded.

Muscle weakness was defined as MMT score < 5/5. Formal MMT was tested for all muscles groups listed in the Clinical GENI for 12/12 participants; however formal MMT was not tested for hip extension in one participant and right ankle DF in one participant due to SMC impairment and joint contracture. In these cases, a score was assigned for analysis based on single leg bridge (score of 3) for hip extensors and ankle DF motion in swing (score of 2) for ankle DF. For MMT score with “+” or “–,”a fractional score of ±0.25 was added to the numeric MMT score for analysis. PROM was tested using goniometry and impairments were identified based on the minimal ROM required for normal gait. Short ankle plantarflexors (PF) was noted if ankle DF with knee extended was ≤ 0°. Short knee flexors were noted if PROM knee extension was ≥5° of knee flexion. Short hamstrings were noted if straight leg raise (SLR) was < 60°. Short hip flexors were noted if hip extension was < 0°. Spasticity was considered present if R2-R1 was ≥5°. Impaired SMC was considered if SCALE score < 10 and/or individual joint score was < 2/2.

### Data analysis

This study assessed validity of the Clinical GENI tool (Figure 1) by evaluating percent agreement between presence of gait abnormalities identified with the observational Clinical GENI tool vs. 3D gait kinematics, based on the criteria listed in the Clinical GENI tool (Figure 1B in Supplemental material). False positives (FP) and false negatives (FN) were reported with FP defined if gait abnormality was identified on video but not on kinematic data, FN were defined if gait abnormality was not identified on video but was on kinematic data. Statistical analysis was performed using SPSS software, version 30. The sample was relatively small (*n* = 12, 24 limbs) and included data with normal and non-normal distributions, identified using Shapiro-Wilk test, therefore both parametric and non-parametric tests were performed. Significance of ordinal data was determined using non-parametric test. Severity of neuromuscular impairments listed in the Clinical GENI tool was compared with and without the gait abnormality identified based on criteria listed in the Clinical GENI tool (Figure 1B in Supplemental material), using the one-tailed parametric *t*-test and non-parametric Mann–Whitney U test (*p* < 0.05) (Table 2). In addition, the correlation between severity of the neuromuscular impairment and severity of gait abnormality based on kinematic data was assessed using the one-tailed non-parametric Spearman Correlation (*p* < 0.05) (Table 3). All gait abnormalities had kinematic data except for forefoot/flatfoot IC which was based exclusively on observational video analysis.

**Table 2:** Severity of neuromuscular impairment with and without gait abnormality.

		**Initial contact gait abnormality**										**Single limb stance gait abnormality**									

		**Forefoot/flatfoot IC**					**Increased knee flexion IC**					**Increased knee flexion SLS**					**Increased hip flexion SLS**				
**Neuromuscular impairment**	**Physical exam**	**No Abn (*****n*** = **8)**	**Gait Abn (*****n*** = **16)**	***t***, ***p*****-value**	**MW U**, ***p*****-value**	**95% CI** wicth 0.5pt	**No Abn (*****n*** = **8)**	**Gait Abn (*****n*** = **16)**	***t***, ***p*****-value**	**MW U**, ***p*****-value**	**95% CI** wicth 0.5pt	**No Abn (*****n*** = **16)**	**Gait Abn (*****n*** = **8)**	***t***, ***p*****-value**	**MW U**, ***p*****-value**	**95% CI** wicth 0.5pt	**No Abn (*****n*** = **14)**	**Gait Abn (*****n*** = **10)**	***t***, ***p*****-value**	**MW U**, ***p*****-value**	**95% CI**
Weak ankle PF	MMT ankle PF					wicth 0.5pt					wicth 0.5pt	2.63	1.5	3.07, *p =* 0.003	20.0, *p =* 0.006	0.365, 1.885 wicth 0.5pt	2.64	1.70	2.57, *p =* 0.009	31.0, *p =* 0.022	0.181, 1.705
Weak ankle DF	MMT ankle DF	5.00	4.22	3.76, *p =* 0.001	24.0, *p* = 0.013	0.338, 1.224 wicth 0.5pt					wicth 0.5pt					wicth 0.5pt					
Short ankle PF, knee flex	PROM ankle DF, knee flex°					wicth 0.5pt					wicth 0.5pt					wicth 0.5pt					
Short ankle PF, knee ext	PROM Ankle DF, knee ext°	1.25	−7.81	2.36, *p =* 0.014	30.0, *p* = 0.038	1.086, 17.039 wicth 0.5pt					wicth 0.5pt					wicth 0.5pt					
Spasticity of Gastroc	Spasticity, Gastroc R2-R1 °	3.13	3.44	−0.11, *p =* 0.455	47.5, *p =* 0.320	−6.001, 5.376 wicth 0.5pt	0.63	4.69	−2.14, *p =* 0.023	42.0, *p =* 0.192	−8.051, −0.073 wicth 0.5pt					wicth 0.5pt					
Weak knee extensors	MMT knee ext					wicth 0.5pt	4.81	4.44	2.02, *p =* 0.028	34.0, *p =* 0.070	−0.009, 0.759 wicth 0.5pt	4.73	4.22	3.05, *p =* 0.003	25.0, *p =* 0.016	0.165, 0.867 wicth 0.5pt	4.70	4.38	1.78, *p =* 0.044	43.0, *p =* 0.122	−0.053, 0.696
Short knee flexors	PROM knee ext°					wicth 0.5pt	3.13	−4.69	2.68, *p =* 0.007	34.0, *p =* 0.070	1.747, 13.878 wicth 0.5pt	2.50	−11.25	3.39, *p =* 0.005	16.5, *p =* 0.002	4.294, 23.206 wicth 0.5pt					
Short hamstrings	PROM, straight leg raise°					wicth 0.5pt	51.25	45.63	1.03, *p =* 0.158	46.5, *p =* 0.291	−5.752, 17.002 wicth 0.5pt	52.50	37.50	3.25, *p =* 0.002	17.0, *p =* 0.003	5.427, 24.573 wicth 0.5pt					
Weak hip extensors	MMT hip ext					wicth 0.5pt					wicth 0.5pt	3.94	3.28	2.25, *p =* 0.017	33.0, *p =* 0.061	0.051, 1.261 wicth 0.5pt	4.02	3.30	2.67, *p =* 0.007	34.0, *p =* 0.036	0.160, 1.276
Short hip flexors	PROM hip ext°					wicth 0.5pt					wicth 0.5pt					wicth 0.5pt	3.93	−13.00	6.70, *p < * 0.001	4.0, *p < * 0.001	11.690, 22.167
Impaired selective motor control	SCALE	7.88	4.50	4.34, *p < * 0.001	18.0, *p =* 0.004	1.763, 4.987 wicth 0.5pt	7.50	4.69	3.22, *p =* 0.002	23.5, *p =* 0.011	1.002, 4.623 wicth 0.5pt					wicth 0.5pt					
Weak ankle PF	MMT ankle PF	1.80	2.37	−1.15, *p =* 0.131	32.5, *p =* 0.297	−1.592, 0.455 wicth 0.5pt					
Weak ankle DF	MMT ankle DF					wicth 0.5pt	4.72	3.75	2.16, *p =* 0.039	24.0, *p =* 0.047	−0.149, 2.094 wicth 0.5pt
Short ankle PF, knee flex	PROM ankle DF, knee flex°					wicth 0.5pt	9.72	4.17	0.80, *p =* 0.251	40.5, *p =* 0.378	−8.801, 19.912 wicth 0.5pt
Short ankle PF, knee ext	PROM ankle DF, knee ext°					wicth 0.5pt					
Spasticity of gastroc	Spasticity, gastroc R2-R1°					wicth 0.5pt					
Weak knee extensors	MMT knee ext					wicth 0.5pt					
Short knee flexors	PROM knee ext°					wicth 0.5pt					
Short hamstrings	PROM, straight leg raise°					wicth 0.5pt					
Weak hip extensors	MMT hip ext					wicth 0.5pt					
Short hip flexors	PROM hip ext°					wicth 0.5pt					
Impaired selective motor control	SCALE	5.00	5.79	−0.65, *p =* 0.262	41.0, *p =* 0.679	−3.315, 1.736 wicth 0.5pt					

Parametric *T*-Test and Mann–Whitney *U*-Test performed to determine relationship between severity of neuromuscular impairment present in limbs identified with and without the gait abnormality.

Gait Abn, Gait Abnormality; No Abn, No Gait Abnormality; MW U, Mann–Whitney *U*-Test; CI, Confidence Interval; IC, Initial Contact; SLS, Single Limb Stance; DF, Dorsiflexion; PF, Plantarflexion.

°Degree.

**Table 3:** Severity of neuromuscular impairment and severity of gait abnormality.

		**Initial contact gait abnormality**		**Single limb stance gait abnormality**				**Pre-swing gait abnormality**		**Mid-swing gait abnormality**	
**Neuromuscular impairment**	**Physical exam**	**Increased knee flexion IC**, ***n*** = **16**		**Increased knee flexion SLS**, ***n*** = **8**		**Increased hip flexion SLS**, ***n*** = **10**		**Reduced ankle PF**, ***n*** = **19**		**Increased ankle PF**, ***n*** = **6**	
		**Spearman Correlation**		**Spearman Correlation**		**Spearman Correlation**		**Spearman Correlation**		**Spearman Correlation**	
		ρ, ***p*****-value**	**95% CI**	ρ, ***p*****-value**	**95% CI**	ρ, ***p*****-value**	**95% CI**	ρ, ***p*****-value**	**95% CI**	ρ, ***p*****-value**	**95% CI**
Weak ankle PF	MMT ankle PF			−0.618, *p* = 0.001	−0.815, −0.339	−0.476, *p* = 0.009	−0.748, −0.147	0.160, *p* = 0.227	−0.282, 0.570		
Weak ankle DF	MMT ankle DF									−0.504, *p* = 0.006	−0.827, −0.082
Short ankle PF, knee flex	PROM ankle DF, knee flex									−0.230, *p* = 0.140	−0.572, 0.179
Short ankle PF, knee ext	PROM ankle DF, knee ext										
Spasticity of gastroc	GS spasticity, R2-R1	0.159, *p* = 0.229	−0.220, 0.498								
Weak knee extensors	MMT knee ext	−0.493, *p* = 0.007	−0.779, −0.116	−0.573, *p* = 0.002	−0.849, −0.233	−0.467, *p* = 0.011	−0.777, −0.097				
Short knee flexors	PROM knee ext	−0.578, *p* = 0.002	−0.785, −0.195	−0.684, *p < * 0.001	−0.846, −0.317						
Short hamstrings	Straight leg raise	−0.458, *p* = 0.012	−0.734, −0.090	−0.621, *p* = 0.001	−0.782, −0.348						
Weak hip extensors	MMT hip ext			−0.346, *p* = 0.049	−0.688, 0.058	−0.391, *p* = 0.029	−0.737, 0.001				
Short hip flexors	PROM hip ext					−0.811, *p* < 0.001	−0.898, −0.649				
Spasticity of rectus	Rectus spasticity, R2-R1										
Impaired selective motor control	SCALE	−0.641, *p < * 0.001	−0.862, −0.308					−0.024, *p* = 0.455	−0.438, 0.423		

Spearman Correlation performed to determine the relationship between the severity of the neuromuscular impairment present and the severity of the gait abnormality present in limbs identified to have a gait abnormality.

CI, Confidence Interval; n, number of limbs; IC, Initial Contact; SLS, Single Limb Stance; DF, Dorsiflexion; PF, Plantarflexion; ρ, Spearman rho.

This study was approved by Stanford University Institutional Review Board #29770 and was in accordance with the Declaration of Helsinki.

## Results

A total of 12 participants with spastic CP (GMFCS I and II), mean age 11.25 ± 4.27 years (range 4–17 years) qualified to participate: 5 males and 7 females (Table 1). All patients had been referred by a physician to the gait lab with a diagnosis of CP. Participants included 7 with bilateral CP and 5 with unilateral CP, 4 who had right hemiplegia. Eight participants had no history of LE surgery and 4 participants had history of LE surgery more than 1 year prior to gait analysis.

### Gait abnormalities

All 12 gait abnormalities listed on the Clinical GENI were identified in participants. The mean number of gait abnormalities identified per participant was 10.1 (mean *R*= 5.1, L= 5.0). Fewer gait abnormalities were identified for participants with GMFCS I compared to GMFCS II, and fewer gait abnormalities were identified for participants with unilateral CP compared to bilateral CP (Table 1). The most common gait abnormalities identified on the Clinical GENI were forefoot/flat foot IC (16/24 limbs), knee flexion at IC (19/24 limbs), increased hip flexion in single limb stance (SLS) (12/24 limbs), increased anterior pelvic tilt in SLS (12/24 limbs), and reduced ankle PF in pre-swing (19/24 limbs).

There was strong agreement between presence of gait abnormalities identified on gait kinematics and the observational Clinical GENI tool (Figure 1), both were based on the gait abnormality criteria listed in the Clinical GENI (Figure 1B in Supplemental material). Level of agreement ranged from 83 to 100%. FP were more common than FN. Agreement was as follows: knee flexion at IC 22/24 limbs (FP: 2/2 limbs), ankle PF in SLS 21/24 limbs (FP: 2/3, FN: 1/3), increased ankle DF in SLS 22/24 limbs (FP: 2/2), increased knee flexion in SLS 23/24 limbs (FP: 1/1), knee hyperextension in SLS 21/24 limbs (FP: 3/3), increased hip flexion in SLS 22/24 limbs (FP: 2/2), anterior pelvic tilt in SLS 24/24, ipsilateral trunk sway in SLS 20/24 (FP: 4/4), reduced ankle PF in pre-swing 21/24 limbs (FP: 2/3, FN: 1/3), ankle PF in swing 20/24 limbs (FP: 4/4), and reduced peak knee flexion in swing 24/24 limbs.

### Neuromuscular impairments

Table 1 lists neuromuscular impairments identified on physical exam for the 12 participants. Severity of muscle weakness was highest for ankle PF (mean MMT R: 2.00, L: 2.50), hip abductors (mean MMT R: 3.58, L: 3.50), and hip extensors (mean MMT R: 3.77, L: 3.66). Severity of short muscle length was highest for hip flexors, knee flexors, and ankle PF, limiting PROM of hip extension, knee extension, and ankle DF with the knee extended, although the coefficient of variability were high, respectively. Spasticity of rectus femoris and gastrocnemius also had high coefficients of variability. Mean SCALE scores were R: 5.58, L: 5.67.

Muscle weakness was the most common neuromuscular impairment, identified in 12/12 participants: weak ankle PF (23/24 limbs), hip abductors (23/24 limbs), hip extensors (21/24 limbs), knee extensors (12/24 limbs), and ankle DF (10/24 limbs). Short muscle tendon units were the second most common neuromuscular impairment, identified in 12/12 participants: tight hamstrings (17/24 limbs), reduced knee extension (6/24 limbs), short hip flexors (12/24 limbs), and short ankle PF (21/24 limbs). Spasticity was identified in 7/12 participants: gastrocnemius (8/24 limbs), rectus femoris (5/24 limbs). Impaired SMC was identified in 12/12 participants (22/24 limbs).

Table 2 reports the severity of neuromuscular impairment in those with and without gait abnormalities for the 6 most common gait abnormalities of the 12 gait abnormalities listed on the Clinical GENI (Figure 1). Comparisons were performed for the most common gait abnormalities and their neuromuscular impairments. The severity of neuromuscular impairments was significantly higher in participants with the associated gait abnormality vs. without the majority of comparisons. Significance is highlighted based on normality of data.

Table 3 reports the correlation between severity of neuromuscular impairment and severity of gait abnormalities for the 5 most common gait abnormalities of the 11 gait abnormalities on the Clinical GENI with kinematic data. Significant correlations were found between severity of neuromuscular impairment and severity of associated gait abnormality, in 14/18 comparisons. Significance is highlighted based on normality of data.

## Discussion

The Clinical GENI tool is intended to provide clinicians with an observational video-based gait evaluation that identifies contributing neuromuscular impairments. The Clinical GENI tool can be performed at low cost in any clinical setting, including Medically Underserved Areas (MUA).

Results of the study indicate that for the 12 participants evaluated with the Clinical GENI tool (Table 1), there was strong agreement, 83–100%, between kinematic-based and observational-based gait abnormalities on the Clinical GENI tool, and a narrow difference, < 5°, for the majority of disagreements. The results support the utility of the observational-based Clinical GENI tool. Strict inclusion and exclusion criteria contributed to relatively small sample size; while small, the sample was representative of common gait patterns seen in spastic CP. Further validation and reliability of the Clinical GENI tool can be evaluated in a larger sample size.

As expected, individuals with GMFCS I had fewer gait abnormalities compared with those with GMFCS II and those with unilateral CP had fewer gait abnormalities compared with those with bilateral CP. The 12 gait abnormalities listed on the Clinical GENI tool are common gait abnormalities in individuals with spastic CP (Salem et al., 2024; Papageorgiou et al., 2019; Rethlefsen et al., 2017; Sutherland and Davids, 1993), including forefoot/flatfoot IC, knee flexion at IC, increased hip flexion in SLS, increased anterior pelvic tilt in SLS, and reduced ankle PF in pre-swing. Agreement between observational and kinematic gait data was performed for all 12 gait abnormalities, whereas comparison of severity of neuromuscular impairments with and without gait abnormality was performed for the 6 most common gait abnormalities and severity of neuromuscular impairment in relation with severity of gait abnormality was analyzed for 5 most common gait abnormalities with kinematic gait data. Although anterior pelvic tilt was common, it was not analyzed for neuromuscular impairments in this study.

The severity of neuromuscular impairments was significantly higher in those with gait abnormalities listed in the Clinical GENI in a majority of comparisons (Table 2). These results support the utility and validity of the Clinical GENI tool and indicate that muscle weakness was the most common neuromuscular impairment associated with gait abnormalities. Short muscle-tendon unit and impaired SMC were the next most common impairments associated with gait abnormalities. The severity of muscle spasticity did not differ in those with and without gait abnormality. Although this sample is small, it is representative of the population of individuals with spastic CP GMFCS I and II and indicates the substantial contribution of muscle weakness to gait abnormalities and the importance of addressing weakness in developing targeted interventions to improve gait.

The severity of neuromuscular impairments was significantly correlated to the severity of gait abnormalities listed on the Clinical GENI tool in a majority of comparisons (Table 3). The severity of muscle weakness, short muscle-tendon unit, and impaired SMC were correlated with the severity of increased knee flexion at IC, whereas spasticity was not correlated. These results support the utility and validity of the Clinical GENI tool and suggest a need to delineate the neuromuscular impairments that contribute to specific gait abnormalities, and avoid overestimating the impact of spasticity. As would be expected biomechanically, the severity of ankle PF weakness and severity of knee extensor weakness were both correlated with the severity of increased knee flexion in SLS and severity of increased hip flexion in SLS. In addition, the severity of ankle DF weakness was correlated with the severity of increased ankle PF in mid-swing, as would be expected. These findings also support the utility and validity of the Clinical GENI tool to accurately identify the presence of gait abnormalities and their contributing neuromuscular impairments. The results also highlight the substantial contribution of muscle weakness to gait abnormalities. Identification of gait abnormalities and their contributing neuromuscular impairments can guide individualized treatments to improve gait and function for individuals with spastic CP.

### Neuromuscular impairment contributions to gait abnormalities

#### Forefoot/flatfoot IC

Forefoot/flatfoot IC can result from any of the four neuromuscular impairments. In this population, the severity of weak ankle DF, short ankle PF with knee extended, and impaired SMC were higher for those with vs. without forefoot/flatfoot IC (Table 2). Weak ankle dorsiflexors can reduce ankle DF in swing. Short ankle PF can increase ankle PF at IC, especially as the knee extends for IC. Potentially, spasticity of the gastrocnemius can limit ankle to DF due to the rapid muscle stretch as the knee extends, although we did not see this in this population. Impaired SMC can also limit ankle DF while the knee is extended. While forefoot and flatfoot IC are different, the neuromuscular impairments that potentially lead to these abnormalities are the same, and both gait abnormalities lack the heel-rocker, which can negatively impact forward progression and energy expenditure (Attias et al., 2023; Salami et al., 2024).

#### Knee flexion at IC

Knee flexion at IC can result from any of the four interrelated neuromuscular impairments. In this population, the severity of weak knee extensors, short knee flexors, short hamstrings, and impaired SMC were higher for those with increased knee flexion at IC (Table 2). In addition, severity of weak knee extensors, short knee flexors, short hamstrings, and impaired SMC correlated with increased knee flexion at IC (Table 3). Weak knee extensors can limit knee extension in terminal swing. This is supported by normal electromyography data that shows vastus lateralis activity in terminal swing (Nene et al., 2004). Full knee extension can also be limited by short knee flexors (Arnold et al., 2006). Potentially, spasticity of the knee flexors, the gastrocnemius or hamstrings, may limit knee extension while the hip is flexed and ankle is DF, although we did not see this in this population. In addition, impaired SMC limits the ability of the knee to extend while the hip remains flexed at IC, as was found in this study.

#### Ankle PF in single limb stance (SLS)

Ankle PF in SLS can result from short ankle PF, however this was not a common gait abnormality in this population and therefore was not analyzed here. Spasticity of the gastrocnemius may also contribute to ankle PF in SLS due to rapid stretch of the gastrocnemius during the ankle rocker or if there is forefoot IC causing rapid loading of the gastrocnemius. Impaired SMC can contribute to ankle PF in SLS as the hip and knee extend. Increased muscle co-activation of knee extensors and gastrocnemius has been identified in children with CP during gait which can be related to impaired SMC and contribute to ankle PF in SLS (Ippersiel et al., 2024; Rose et al., 1999).

#### Increased ankle DF in SLS

Increased ankle DF in SLS can result from ankle PF weakness failing to restrain forward progression of the tibia (Attias et al., 2023). Ankle DF in SLS is controlled by ankle PF eccentric contraction; in the presence of ankle PF weakness there is less control of this forward progression. Increased ankle DF in SLS was not a common gait abnormality in this population, therefore was not analyzed.

#### Increased knee flexion in SLS

Increased knee flexion in SLS can result from weakness in hip extensors, knee extensors, and ankle PF. In this population, the severity of weak ankle PF, weak knee extensors, and short knee flexors and hamstrings was higher in those with increased knee flexion in SLS (Table 2). The severity of weak ankle PF, weak knee extensors, short knee flexors and hamstrings, and weak hip extensors correlated with the severity of increased knee flexion in SLS (Table 3). Weak hip extensors limit full hip and knee extension in stance. Weak knee extensors also limit full knee extension in stance, which increases the knee extensor moment and places further demand on the quadriceps. Weakness of ankle PF can contribute to increased ankle DF, which further contributes to increased knee flexion. Short knee flexors also limit full knee extension in SLS.

#### Knee hyperextension in SLS

Knee hyperextension in SLS can result from weak knee extensors. Rapid knee hyperextension can be a protective mechanism to reduce risk of knee buckling when flexed. This can occur as early as loading response, but will continue into SLS. Short ankle PF can contribute to knee hyperextension, as increased ankle PF can move the ground reaction force anterior to the knee in early stance, which promotes knee hyperextension. Knee hyperextension in SLS was not a common gait abnormality in this population, therefore was not analyzed.

#### Increased hip flexion in SLS

Increased knee flexion in SLS can result from weakness of hip extensors, knee extensors, ankle PF. In this population, the severity of weak ankle PF, weak knee extensors, weak hip extensors, and short hip flexors were higher in those with increased hip flexion in SLS (Table 2). Further, severity of weak ankle PF, weak knee extensors, weak hip extensors, and short hip flexors correlated with severity of increased hip flexion in SLS (Table 3). Weakness of hip extensors limits full hip extension in stance. Weakness of knee extensors contribute to knee flexion in SLS which limits hip extension. Ankle PF weakness contributes to increased ankle DF and knee flexion in SLS and limits hip extension. Short hip flexors also contribute to increased hip flexion.

#### Anterior pelvic tilt in SLS

While some degree of anterior pelvic tilt is present in neurotypical individuals (Suits, 2021), increased anterior pelvic tilt in SLS can be caused by weak hip extensors (or abdominals). Short hip flexors can also cause increased anterior pelvic tilt. Although anterior pelvic tilt was common, it was not analyzed for neuromuscular impairments in this study.

#### Ipsilateral trunk sway in SLS

Ipsilateral trunk sway in SLS can be caused by weakness of the ipsilateral, stance limb, hip abductors (Krautwurst et al., 2013). While in SLS the hip abductors act to stabilize the pelvis. Ipsilateral sway of the trunk brings the center of mass closer to the hip joint center, reducing the length of the lever arm, hip joint torque and the demand on the muscle. Although ipsilateral trunk sway in SLS was common, it was not analyzed for neuromuscular impairments in this study.

#### Reduced ankle PF in pre-swing

Reduced ankle PF in pre-swing is often related to weak ankle PF and reduced ankle PF power generation in pre-swing, though this was not the case in this population (Tables 2, 3). Impaired SMC can also contribute to reduced ankle PF in pre-swing: as the hip and knee are beginning to progress toward flexion the ankle is progressing toward PF which is challenging to isolate if SMC impairment is present. Pre-swing is an important preparation phase for swing; pre-swing gait abnormalities may contribute to reduced knee flexion in swing and reduced foot-floor clearance.

#### Ankle PF in initial-mid swing

Ankle PF in initial-mid swing is often due to weak ankle DF. Rapid contraction of the ankle DF in swing is required to achieve foot-floor clearance. Similarly, in this population, the severity of weak ankle DF was higher for those with ankle PF in initial-mid swing (Table 2). The severity of both weak ankle DF and short ankle PF correlated with the severity of ankle PF in mid-swing (Table 3). Of note, PROM measurement of ankle DF with the knee flexed may be more important for understanding the impact of short ankle PF in swing, as the knee is flexed during this phase.

#### Reduced peak knee flexion in initial-mid swing

Reduced peak knee flexion in initial-mid swing may result from weak ankle PF. Generation of ankle PF power in pre-swing is critical for segmental kinetic energy of the limb during swing and contributes to swing limb acceleration (Zelik and Adamczyk, 2016; Choi et al., 2023). Weakness of ankle PF can limit power generation. Short hip flexors can also contribute to reduced peak knee flexion in swing. The hip flexors store elastic energy in pre-swing and act like a spring (Koussou et al., 2021). If the hip is unable to achieve sufficient extension in stance this can impact the elastic energy storage and pre-swing mechanics, ultimately impacting swing gait pattern. That pre-swing can have a significant impact on swing mechanics, highlights the contribution of the kinetic chain to gait abnormalities. Potentially, spasticity of the rectus femoris can also contribute to reduced peak knee flexion in swing as the knee rapidly flexes. However, hip flexion reduces stretch to the rectus femoris, which is normally active in initial swing, reducing the likelihood of this limiting knee flexion. Reduced peak knee flexion in initial-mid swing was not common in this population, therefore was not analyzed.

Limitations of the study include a small sample size of 12 individuals, 24 limbs; although this sample was representative of individuals with spastic CP and had strict inclusion criteria. A larger scale analysis is needed to confirm these findings and further investigate associations between neuromuscular impairments and 12 gait abnormalities listed on the Clinical GENI tool. Future studies can also investigate the impact of neuromuscular impairments on trunk and pelvic motion during gait.

## Conclusion

The Clinical GENI tool provides clinicians with the ability to more accurately and easily identify gait abnormalities and the contributions of neuromuscular impairments, in any clinical setting. This can improve access to gait analysis and guide individualized and effective interventions. Advantages of this tool include no additional cost, efficiency of use, and no need of additional equipment, other than video. The results of this study support the clinical utility and validity of the Clinical GENI tool.

## Data Availability

The raw data supporting the conclusions of this article will be made available by the authors, without undue reservation.
