# Supplementary figures and images for: Clinical Gait Evaluation with Neuromuscular Impairments (Clinical GENI) for spastic cerebral palsy
Source: Front Hum Neurosci. 2025 Sep 17;19:1637164. doi: 10.3389/fnhum.2025.1637164 (PMC12484064; doi:10.3389/fnhum.2025.1637164)

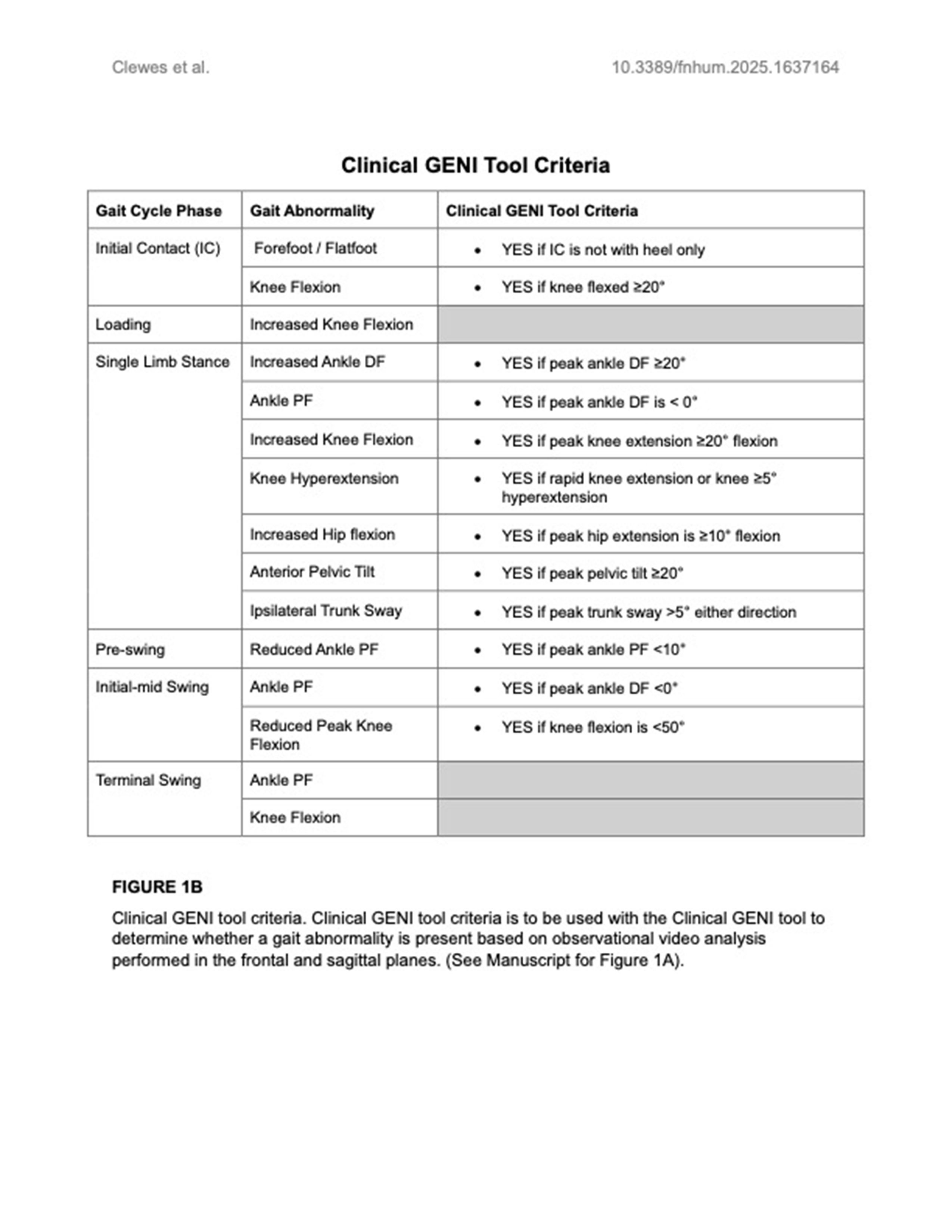

Supplement: Supplementary file 1 [file Image_1.jpg]
